# The mTOR kinase inhibitor Everolimus decreases S6 kinase phosphorylation but fails to reduce mutant huntingtin levels in brain and is not neuroprotective in the R6/2 mouse model of Huntington's disease

**DOI:** 10.1186/1750-1326-5-26

**Published:** 2010-06-22

**Authors:** Jonathan H Fox, Teal Connor, Vanita Chopra, Kate Dorsey, Jibrin A Kama, Dorothee Bleckmann, Claudia Betschart, Daniel Hoyer, Stefan Frentzel, Marian DiFiglia, Paolo Paganetti, Steven M Hersch

**Affiliations:** 1 MassGeneral Institute for Neurodegenerative Disease and Harvard Medical School, Charlestown, MA, USA; 2 Novartis Institutes for Biomedical Research, Neuroscience Discovery, Novartis Pharma AG, Basel, Switzerland; 3 Department of Veterinary Science and Graduate Neuroscience program, University of Wyoming, Laramie, WY, USA

## Abstract

**Background:**

Huntington's disease (HD) is a progressive neurodegenerative disorder caused by a CAG repeat expansion within the huntingtin gene. Mutant huntingtin protein misfolds and accumulates within neurons where it mediates its toxic effects. Promoting mutant huntingtin clearance by activating macroautophagy is one approach for treating Huntington's disease (HD). In this study, we evaluated the mTOR kinase inhibitor and macroautophagy promoting drug everolimus in the R6/2 mouse model of HD.

**Results:**

Everolimus decreased phosphorylation of the mTOR target protein S6 kinase indicating brain penetration. However, everolimus did not activate brain macroautophagy as measured by LC3B Western blot analysis. Everolimus protected against early declines in motor performance; however, we found no evidence for neuroprotection as determined by brain pathology. In muscle but not brain, everolimus significantly decreased soluble mutant huntingtin levels.

**Conclusions:**

Our data suggests that beneficial behavioral effects of everolimus in R6/2 mice result primarily from effects on muscle. Even though everolimus significantly modulated its target brain S6 kinase, this did not decrease mutant huntingtin levels or provide neuroprotection.

## Background

Huntington's disease (HD) is a progressive neurodegenerative disorder caused by a glutamine-encoding CAG repeat expansion within the huntingtin gene [1]. Neurodegeneration is most prominent within striatum and neocortex and results in abnormal movements, cognitive decline and psychiatric symptoms. Mutant huntingtin misfolds and accumulates as soluble and insoluble aggregated species primarily in neurons.

Macroautophagy is a lysosomal-dependent process that mediates the turnover of organelles and misfolded proteins that are too large to be degraded by the ubiquitin proteosomal system [2,3]. Steps involve biochemical induction, the sequestering of cytoplasmic fragments into double-membrane bound autophagic vacuoles, subsequent fusion with lysosomes and degradation within autolysosomes [4]. The process involves the coordinated expression and regulation of many core and autophagy-related [5] as well as lysosomal proteins [6]. There is activation of macroautophagy in HD models [3,7]. Macroautophagy is also involved in the pathogenesis of Parkinson's, Alzheimer's and prion diseases [8-10].

Promoting clearance of mutant huntingtin (mhtt) by induction of macroautophagy is one approach for treating human HD [7,11]. Everolimus (formerly called RAD001) is an inhibitor of mammalian target of rapamycin (mTOR), a protein that is part of an intra-cellular signaling pathway regulating cell metabolism. Everolimus, like rapamycin, inhibits the kinase activity of the raptor-mTOR complex (mTORC1) by binding to the protein FKBP-12, which forms an inhibitory complex with mTOR [12,13]. mTOR kinase is a cytosolic protein that receives inputs from nutrient signaling pathways and is an inhibitor of macroautophagy [14,15]. Everolimus inhibition of mTOR kinase promotes macroautophagy in a number of model systems [16,17]. mTOR-kinase-independent macrophagy inducers have also been identified [9,11] and these may offer an alternative pathway to modulate autophagy. However, the class of mTOR-kinase-inhibiting drugs is well characterized and in clinical use for their anti-neoplastic and anti-solid organ graft rejection effects [18,19]. These compounds would offer advantages of availability and rapid progression into clinical trials if found to have significant beneficial effects in HD models.

The goal of this study was to evaluate the effect of everolimus in the R6/2 transgenic mouse model of HD. These mice express the exon-1 encoded fragment of mutant huntingtin under the control of the huntingtin promoter which results in protein expression in brain and skeletal muscle [20]. We found that everolimus retarded declines in motor improvements. In brain, everolimus inhibited phosphorylation of the mTOR kinase target protein S6 kinase, but did not decrease mutant huntingtin levels, or decrease brain and neuronal atrophy. However, in skeletal muscle everolimus significantly decreased levels of soluble mutant huntingtin protein. While our data demonstrates a beneficial effect of everolimus in R6/2 HD mice, we could not demonstrate neuroprotection.

## Results

### Pharmacokinetic analysis of everolimus in R6/2 mice

**Figure 1:**
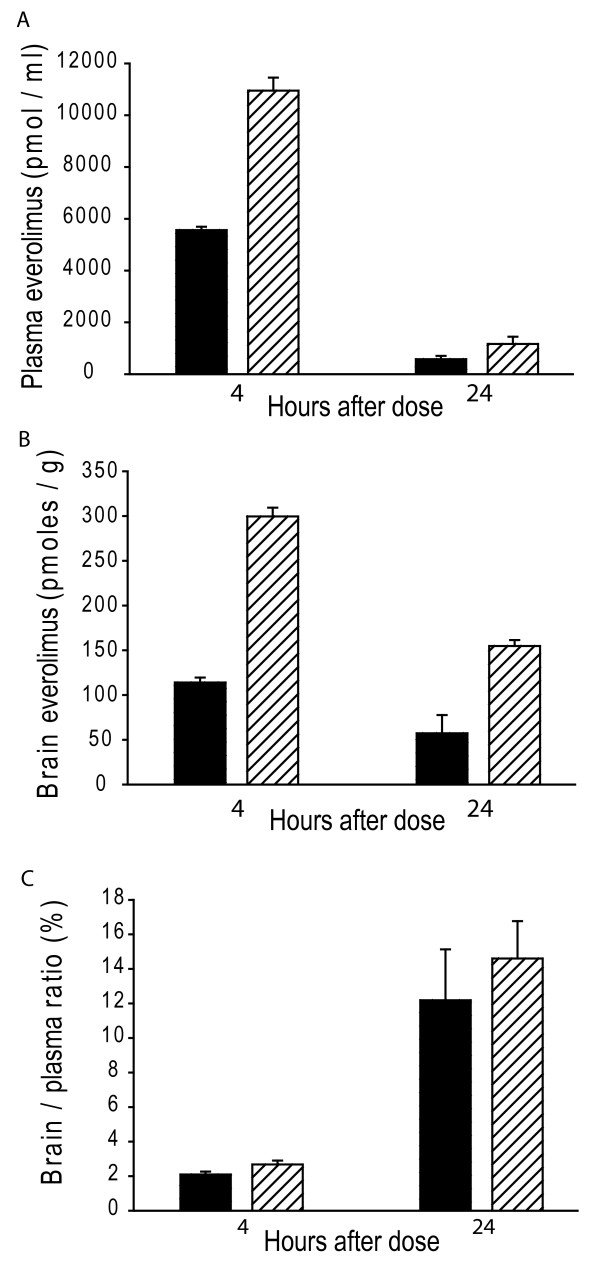
**Everolimus penetrates the blood-brain barrier in R6/2 HD transgenic mice**. Plasma (**A**) and brain (**B**) everolimus concentrations were measured 4 and 24 hours after the last dose following two weeks of treatment (black-bar = 10 μmol/kg, cross-hatched bar = 30 μmol/kg). Brain/plasma ratios (calculated as the ratio of brain to plasma concentration × 100) are higher than the reported [29] level of blood contamination (~6%) at 24, but not at 4 hours (**C**). Shown are mean ± SE. n = 3.

Mice were treated from 6-8 weeks at 10 and 30 μmol/kg. Plasma and brain everolimus was quantified 4 and 24 hours after the last dose. The analytical method used provided robust measures of everolimus in mouse plasma and brain. Limits of quantification were 1.5 pmol/ml plasma or 7.5 pmol/g brain, respectively. Four hours after the last dose mean plasma everolimus concentrations were 5560 and 10950 pmoles/ml at the 10 and 30 μmol/kg doses, respectively (Figure 1A). Corresponding brain concentrations were 113 and 299 pmoles/g (Figure 1B). Twenty-four hours after dosing mean plasma everolimus concentrations were significantly lower at 580 and 1160 pmoles/ml for the 10 and 30 μmol/kg doses, respectively (Figure 1A). Corresponding mean brain concentrations at 24 hours were 57 and 154 pmoles/g (Figure 1B). Brain everolimus was 2-3% of plasma concentration at 4-hours consistent with plasma contamination. However, at 24 hours it was 12-14% indicating significant brain penetration (Figure 1C) (see discussion). Weight loss is a prominent feature of human and mouse HD. Because pilot tolerability experiments in R6/2 HD mice demonstrated weight loss exceeding 10% with the 30 μmol/kg dose (our working definition of maximum tolerated dose) we tested 10 and 20 μmol/kg doses in subsequent experiments. The use of 20 μmol/kg as a maximum dose is further supported by the finding that it resulted in significantly reduced weight gain from 6 weeks of age as compared to vehicle-treated transgenic mice.

### Everolimus decreases brain S6 kinase phosphorylation

**Figure 2:**
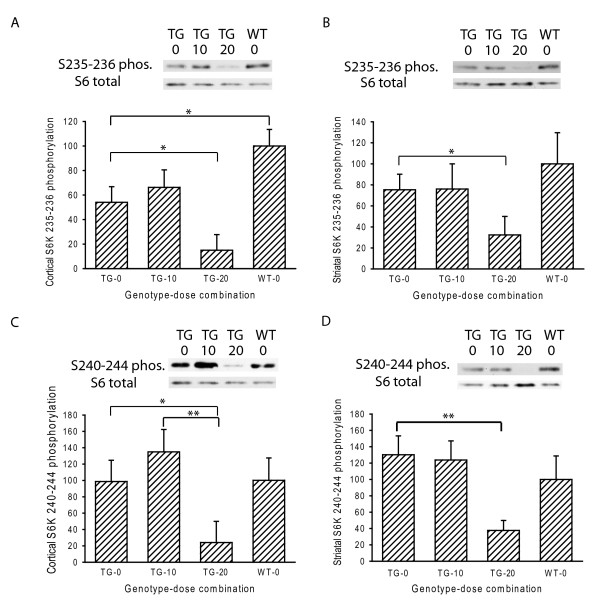
**Mutant huntingtin and everolimus decrease S6 kinase phosphorylation in mouse HD brain**. High (20 μmol/kg) but not low (10 μmol/kg) dose everolimus significantly decreases phosphorylation of S6 kinase at the S235-236 epitopes in cortex (**A**) and striatum (**B**). High-dose everolimus also significantly decreases phosphorylation of S6-kinase at the S240-244 epitopes in cortex (**C**) and striatum (**D**). S6 kinase phosphorylation at S235-236 is decreased in vehicle-treated HD compared to wild-type mice (**A**). Mice were treated from 4-12 weeks and sacrificed 4 hours after the last dose. X-axes show genotype-dose combinations. TG = transgenic, WT = wild-type. n = 10. p-values: * = < 0.05, ** = < 0.01

Our pharmacokinetic analysis indicates brain penetration of everolimus. To determine if the concentration in brain is sufficient to inhibit mTOR kinase we undertook a Western blot analysis of S6 kinase, a direct phosphorylation target of mTOR kinase. We chose to evaluate everolimus by dosing on Mondays, Wednesdays and Fridays for two reasons. First, three doses/week were used when testing the rapamycin and everolimus analog CCI-779 in N171-82Q HD mice [7]. Second, in preliminary experiments a single dose of 30 μmol/kg decreased brain S6 kinase phosphorylation for 24 hours (not shown). Mice were dosed from 4-12 weeks and sacrificed 4 hours after the last dose. S6 kinase phosphorylation at the S235-236 epitope was significantly decreased in cortex of R6/2 compared to wild-type mice (Figure 2A). Twenty, but not 10, μmol/kg everolimus resulted in a significant decrease in S235-236 phosphorylation in cortex and striatum (Figure 2A-B). There was no effect of mutant huntingtin expression on phosphorylation of the S240-244 epitope. Twenty, but not 10, μmol/kg everolimus significantly decreased phosphorylation at this epitope (Figure 2C-D). Our pharmacokinetic studies indicated no brain penetration four hours after dosing (Figure 1C). However, Western blot analysis revealed significant effects on S6 kinase phosphorylation four hours after a final dose comprising a total of 8 weeks treatment (Figure 2). Together, these data suggest that after prolonged treatment brain effects of the 20 μmol/kg dose last up to 48 hours.

### Effect of everolimus on mouse performance and brain pathology

**Figure 3:**
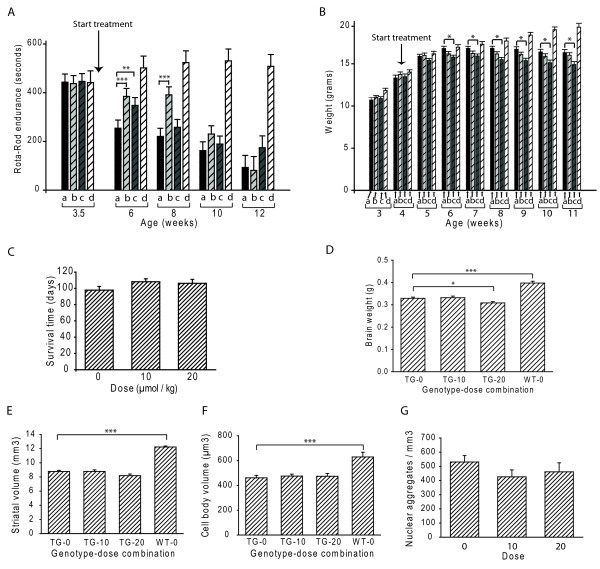
**Everolimus provides early benefit against motor decline in R6/2 HD mice**. **A-B**. Bars: solid black (a) = HD vehicle: cross-hatched; light gray (b) = low dose, dark gray (c) = high dose, white (d) = wild-type vehicle treated. WT to HD vehicle comparison significance bars are omitted for clarity. **A. **Low and high dose everolimus (10 and 20 μmol/kg, respectively) provide early, but not late, benefit against the decline in Rota-rod endurance in R6/2 HD mice. **B**. High dose everolimus has a small negative effect on body weight. Everolimus treatment does not improve measures of survival (**C**), brain weight (**D**), striatal volume (**E**), cell body volume (**F**) and nuclear aggregate density (**G**). Mice were dosed from 4-weeks of age. Shown are means + SE. X-axes show genotype-dose combinations (**D-F**). TG = transgenic, WT = wild type. n = 15-20 for behavior and survival (**A-C**), n = 12 for quantitative pathology (**D-G**). p-values: * = < 0.05

We evaluated the effect of 10 and 20 μmol/kg everolimus on mouse Rota-Rod endurance. Mice were treated from 4 weeks of age. At 6 and 8 weeks of age, 10 μmol/kg everolimus provided significant benefit against the decline in Rota-Rod endurance (Figure 3A). Twenty μmol/kg everolimus provided benefit at 6 weeks only (Figure 3A). Ten μmol/kg everolimus had no effect on body weight, while 20 μmol/kg resulted in a significantly lower, but not progressively declining body weight from 6-weeks (Figure 3B). There was no effect of everolimus on mean survival times (Figure 3C). We sought to determine if the beneficial effect of everolimus is due to a protective effect in brain. R6/2 mice have progressive declines in brain size and increases in mutant huntingtin aggregate burden [21]. Despite the beneficial behavioral effect of everolimus, neither dose was protective against loss of brain mass as measured by brain weight, striatal volume, striatal neuronal cell body volume and nuclear aggregate density (Figure 3D-G). Twenty μmol/kg everolimus resulted in significantly lower brain weight, which is consistent with the lower body weight at this dose (Figure 3D).

### LC3B cleavage

**Figure 4:**
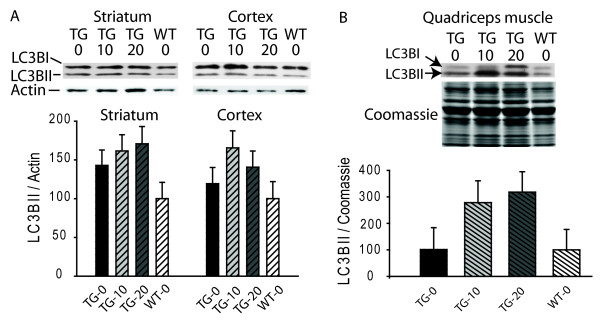
**Everolimus does not affect LC3BII levels in mouse HD brain**. There is no significant effect of everolimus on normalized LC3BII levels in striatum and cortex (**A**). There is a trend towards increased normalized LC3BII levels in muscle of everolimus treated R6/2 mice (global p-value = 0.12) (**B**). Mice were treated from 4-12 weeks and sacrificed 4-hours after the last dose. X-axes show genotype-dose combinations. TG = transgenic, WT = wild-type. n = 10.

Upon synthesis, LC3B is cleaved into the cytosolic protein LC3BI. Activation of macroautophagy leads to conversion of LC3BI into LC3BII, a form that associates with autophagosomes [22]. Both normalized LC3BII levels and the LC3BI: LC3BII ratio (conversion) have been used as measures of macroautophagy activation [22,23]. We therefore measured these parameters in brain and muscle of R6/2 HD mice. LC3B I/II ratios were significantly decreased (increased conversion) in striatum, but not cortex, of R6/2 HD mice as compared to control mice (not shown). However, there was no effect of everolimus on normalized LC3BII levels (Figure 4A) or LC3B conversion (not shown) in R6/2 HD brain. In muscle, there was significantly more variability in Western blot results as compared to brain. However, there was a trend towards increased normalized LC3BII levels in muscle of everolimus treated R6/2 mice (global p-value = 0.12; Figure 4B).

### Everolimus normalizes increased LAMP1 in muscle but not in brain

**Figure 5:**
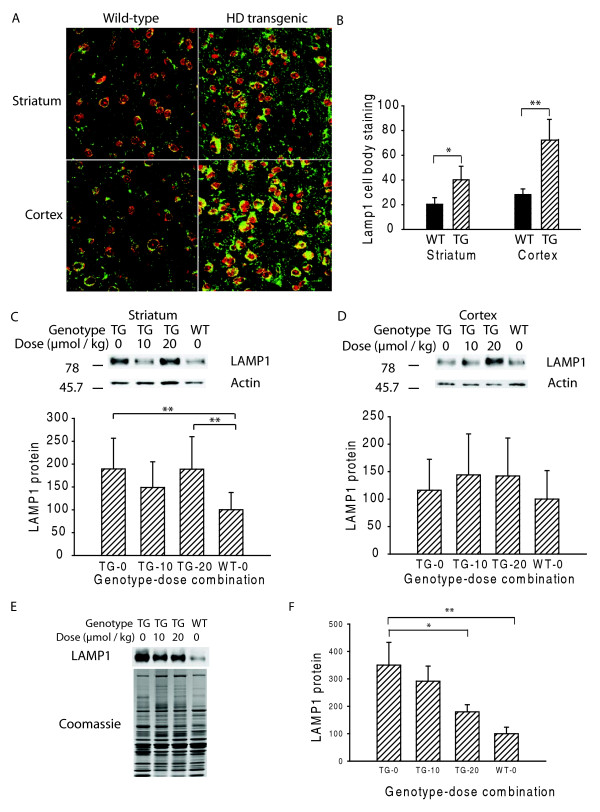
**Everolimus decreases LAMP1 expression in muscle, but not brain**. **A-B**. Lysosome-associated membrane protein 1 (LAMP1) is increased in mouse HD striatal and cortical neurons (green) as measured by quantitative immunofluorescence. Ethidium dimer DNA/RNA counter stain (red). n = 5. **C-D**. There is no effect of everolimus on LAMP1 levels in striatum or cortex as measured by Western blot analysis. **E-F**. LAMP1 is increased in quadriceps femoris muscle; levels are significantly reduced by high-dose everolimus. Shown are means and standard errors. X-axes show genotype-dose combinations (**C, D **and **F**). TG = transgenic, WT = wild-type. n = 8-10. p-values: * = p < 0.05, ** = p < 0.01.

Lysosome-associated membrane protein 1 (LAMP1) is primarily a lysosomal protein [24] that could be a good marker for late steps in the autophagy cascade. We therefore measured LAMP1 protein levels in brain by immunofluorescence and found significant increases in neuronal cell bodies of striatum and cortex of 12-week R6/2 mice (Figure 5A-B). Neuropil LAMP1 staining was not quantified, but also appeared increased in R6/2 mice. Western blot analysis corroborated this finding in striatum, but not cortex. Further, an effect of everolimus on LAMP1 expression in brain was not detected (Figure 5C-D). We evaluated LAMP1 in quadriceps muscle and found that LAMP1 was significantly increased in HD control as compared to wild-type mice. Further, high-dose everolimus significantly decreased LAMP1 close to wild-type levels (Figure 5E-F).

### Everolimus decreases soluble mutant huntingtin levels in muscle but not brain

**Figure 6:**
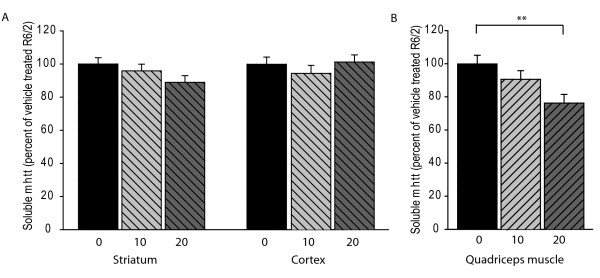
**Everolimus decreases soluble mutant huntingtin levels in muscle, but not brain**. Relative quantification of soluble mutant huntingtin by time-resolved FRET. There is no effect of everolimus on soluble mutant huntingtin levels in striatum and cortex (**A**). Everolimus significantly decreases mean soluble mutant huntingtin in quadriceps muscle (**B**). X-axes = dose (μmoles/kg). ** = p < 0.01, n = 12

Time-resolved Förster resonance energy transfer (time-resolved FRET) is a recently described technique that can quantify soluble mutant huntingtin (mhtt) levels [25]. We used this method to quantify levels of soluble mhtt in muscle and brain. In agreement with our previous results we found that everolimus had no effect on soluble mutant huntingtin levels in striatum or cortex (Figure 6A). This result was corroborated by Western blot analysis that revealed that monomeric mhtt levels were unaltered in occipital cortex of everolimus treated mice (not shown). However, we found that 20 μmol everolimus decreased soluble mutant huntingtin levels in quadriceps muscle (Figure 6B). We then used the recently described Agarose gel electrophoresis for resolving aggregates (AGERA) biochemical assay to quantify mutant huntingtin aggregates [26]. In agreement with the quantitative pathology data (Figure 3G) there was no effect of everolimus on brain aggregates (see Additional file 1: Figure S1A-B). While everolimus decreased soluble mutant huntingtin levels in muscle of R6/2 mice (Figure 6B), there was no effect of everolimus on aggregated huntingtin as measured by AGERA (see Additional file 1: Figure S1C-D).

## Discussion

R6/2 HD mice have been used extensively in pre-clinical drug trials and numerous compounds, including one that promotes macroautophagy, have shown neuroprotective effects [9,27]. We studied the effect of everolimus, a *O*-(2-hydroxyethyl) chain substitution of rapamycin [28] in these mice. We hypothesized that everolimus would decrease mutant huntingtin levels in brain and have neuroprotective effects as measured by decreased brain atrophy. To verify that everolimus could interact with and inhibit its target, brain mTOR kinase, we performed a pharmacokinetic study and also evaluated the phosphorylation state of the mTOR target, S6 kinase. Our results demonstrate that everolimus slowly penetrates brain at doses of 10 and 30 μmol/kg (Figure 1). Brain: plasma ratios 4 hours after a final dose were 2-3% which is consistent with plasma contamination (Figure 1C). However, at 24 hours, brain: plasma ratios were 12-14% which is significantly greater than mouse fore-brain vascular volume of ~6% indicating significant brain penetration [29]. Brain everolimus concentrations were in the range 50-180 nM at 24 hours. The IC_50 _of everolimus in cell proliferation assays is in the sub-nano molar range [30] which is consistent with the concentrations we found in brain being sufficient to strongly inhibit mTOR kinase. To address this, we used S6 kinase phosphoepitope Western blot analysis. We reduced our high dose from 30 to 20 μmol/kg at this point because of body weight loss following prolonged treatment at the 30 μmol/kg dose. Even though we did not repeat the pharmacokinetic analysis using the 20 μmol/kg dose our Western blot analysis clearly shows decreased S6 kinase phosphorylation at the 20 but not the 10 μmol/kg dose, confirming brain penetration at the higher dose used (Figure 2). Decreased S6 kinase phosphorylation in R6/2 HD cortex at the S235-236 epitope, as compared to wild-type mice, is consistent with findings in the N171-82Q HD mouse [7].

Despite demonstrating penetration of everolimus into brain and modulation of its target, we were unable to demonstrate any protective effects in R6/2 HD mouse brain as determined by a detailed quantitative neuropathology study (Figure 3D-G) and using three independent methods to quantify mutant huntingtin levels (Figures 3G, 6, see also Additional file 1: Figure S1) all at 12 weeks of age. Our body weight data (Figure 3B) demonstrate that the high (20 μmol/kg) dose we used in our efficacy study was the maximum tolerated dose using our working definition of a 10% loss of body weight over the study period. As brain weights were slightly but significantly lower in the 20 μmol/kg versus placebo group (Figure 3D), this further suggests that doses higher than that used would not have shown beneficial effects. The three times a week dosing paradigm that we used has been reported previously for the closely related rapamycin analog CCI-779 in a study in N171-82Q HD mice [7]. The suitability of this dosing frequency is also supported by our own data in which mice sacrificed four hours after the last dose of an eight week dosing study had decreased brain S6 kinase phosphorylation (Figure 2) even though everolimus did not enter brain 4 hours after a final dose in the pharmokinetic study (Figure 1). This suggests that with repeated, three times a week, dosing everolimus has a prolonged effect on S6 kinase phosphorylation. Taken together, our results suggest that failure to find a neuroprotective effect of everolimus in R6/2 HD mice was not due to insufficient inhibition of brain mTOR kinase activity.

Muscle is a target of mutant huntingtin in mouse and human HD [31,32]. We found that everolimus demonstrated an early protective effect on Rota-rod performance that was stronger for the low (10 μmol/kg) dose group (Figure 3A). As there was no effect of this low dose on brain S6 kinase phosphorylation (Figure 2) we reasoned that a beneficial effect of everolimus in muscle could explain why the 10 μmol/kg group performed better than the 20 μmol/kg group on Rota-Rod analysis. We did not measure S6 kinase phosphorylation in muscle. However, both mTOR and S6 kinase are expressed in muscle [33]. Given the sub-nanomolar IC_50 _of everolimus for mTOR kinase and expected high penetration of everolimus into muscle, we would expect strong suppression of S6 kinase phosphorylation. Instead, we measured mutant huntingtin levels in everolimus treated R6/2 mice at 12 weeks by FRET. We found significantly decreased soluble mutant huntingtin at the high dose and a trend towards decreased levels at the low dose (Figure 6). There was no effect on aggregated huntingtin levels (see Additional file 1: Figure S1 C-D). Better Rota-rod performance in the low dose group (Figure 3A) could be related to the high dose having both beneficial and toxic effects (Figure 3B). Therefore, our data are consistent with Rota-Rod effects of everolimus being, at least in part, due to beneficial effects in skeletal muscle. We cannot rule out the possibility of a transient suppression of mutant huntingtin levels in brain occurring in the 6-8 week period contributing to behavioral benefits. However, as several compounds demonstrate prolonged protective activity in R6/2 brain [34], including one that promotes autophagy [9,27], this would suggest that even if everolimus decreased brain mutant huntingtin levels transiently that mTOR kinase inhibition is not as effective as modulation of other therapeutic targets in R6/2 mice.

LAMP1 is a type I transmembrane glycoprotein and a marker of lysosomes and autolysosomes [24], organelles critical for downstream steps of the autophagy cascade. While not a core macroautophagy protein, LAMP1 is a lysosome marker and therefore should reflect activity within the terminal clearance steps of the autophagic cascade. We evaluated LAMP1 in our study to determine if levels are increased in R6/2 HD mice and to determine the effect of everolimus. As expected, we found that LAMP1 protein levels were increased in muscle and brain (Figure 5) of R6/2 as compared to wild-type mice. Everolimus had a significant effect on LAMP1 in muscle, but not brain. In muscle, high dose everolimus decreased LAMP1 towards wild-type levels and there was a trend towards a decrease with the low dose (Figure 5E-F). This result was unexpected and the reasons are not clear. We speculate that everolimus may promote clearance of autolysosomes. Further studies are clearly needed to better understand the mechanism(s) by which everolimus decreases mutant huntingtin and LAMP1 levels in muscle.

CCI-779 is a rapamycin dihydoxymethyl propionic acid ester [28] that has been shown to demonstrate behavioral benefits and decrease aggregate density in N171-82Q HD mice [7]. Our results using everolimus, a related rapamycin derivative in the R6/2 HD model demonstrate a different effect. While our data confirms entry of everolimus into brain, as measured by direct chemical analysis and S6 kinase phosphorylation levels, we could not demonstrate decreased neurodegeneration or brain mutant huntingtin levels. The difference between our findings and that of Ravikumar et al (2004) could be for a number of reasons. R6/2 HD mice have a more aggressive phenotype than the N171-82Q HD mice used in the CCI-779 study [21,35]. Factors such as longer polyglutamine expansion in R6/2 compared to N171-82Q mice could also be important.

## Conclusions

Our findings suggest that mTOR kinase inhibition in R6/2 HD mice using everolimus has, at most, a minimal effect on disease progression in brain. Everolimus effectively modulated brain S6 kinase, which is upstream of macroautophagy induction, but had no effect on mutant huntingtin levels. Everolimus did decrease soluble mutant huntingtin levels in muscle. While the exact mechanisms by which this occurs were not fully established, our data indicates that a beneficial effect of everolimus in muscle is a likely mechanism for the protective behavioral effects observed in our HD mice. Given the discrepancy between our findings using everolimus in R6/2 mice and Ravikumar et al [7] using CCI-779 in N171-82Q mice, side-by-side testing of these molecules in both R6/2 and N171-82Q mice, and perhaps as well in a full-length huntingtin mouse model, would provide additional insight into the value of this class of molecule as a treatment for HD.

## Materials and methods

### Mouse studies

R6/2 mice were maintained by crossing R6/2 males (available from The Jackson Laboratory, Bar Harbor, ME) with C57BL/6 X CBA F_1 _females. Tail tips were obtained at 14 days. Tail DNA CAG expansion sizes of male breeder mice were in the range 120-130, as determined by Laragen Inc. Mice were weaned at 22 days, and assigned to treatment groups 1-2 days later. Systematic assignment to treatment groups was used to minimize the effects of body weight variability, litter and paternal effects. Everolimus was prepared as described [36]. Mice were female and were dosed by gavage on Mondays, Wednesdays and Fridays (see discussion). Body weights were measured weekly and used to adjust doses.

### Pharmacokinetic analysis

One-hundred μl plasma was extracted three times in 500 μl ethyl acetate. Extracts were dried under a stream of nitrogen before re-dissolving in 100 μl acetonitrile. Brains were weighed then homogenized in water (1:5 w/v) using an Ultra-Turrax^® ^Mod T8 for 30 seconds. Two 100 μl aliquots of each homogenate were extracted thrice with 500 μl ethyl acetate, then processed as for plasma. Calibration standards were prepared by supplementing 100 μl of mouse plasma (from untreated animals) with everolimus and the analysis quality monitored by routine use of an external standard. For HPLC separation, a Nucleosil CC-125/2 C4 reversed phase column (Macherey & Nagel, Oensingen, Switzerland) under isocratic conditions using 60% acetonitrile and 0.05% formic acid in H_2_O (v/v) with a column temperature of 40°C was used. The flow rate was 0.35 ml/minute and sample injection volume was 10 μl. Retention times were 1.6 and 2.2 minutes for everolimus and NVP-BDF461, respectively. Column efflux was introduced directly into the ion source of a Micromass Platform II LC detector (single quadrupole). The MS conditions were as follows: ionization APCI negative polarity, corona voltage set to 3.2 kV, fragmentor voltage (cone) 50 V, source temperature 350°C. Quantitative analysis was performed by selected ion recording over the de-protonated molecular ion [M+H^-^] of everolimus (m/z 956.8 ± 0.5). Peaks were integrated using MassLynx (Micromass). Two independent extractions were analyzed per animal. Standard curves were prepared by spiking plasma and brain homogenates originating from untreated animals with five concentrations of everolimus as external standard. A second set of standards in acetonitrile was directly analyzed to estimate extraction yield. A linear calibration was calculated for each analytical batch from the ratio between calibrant and internal standard and the calibrant in spiked plasma or brain samples. Regression was performed using Origin^® ^software. Unknown concentrations were calculated from the calibration parameters obtained with extracted samples containing internal standard.

### Western blot analysis

Antibodies used were: mutant huntingtin (MAB5492-Chemicon), actin (AC40-Sigma), LC3B (Novus Biologicals) and LAMP1 (BioLegend). Total S6 (54D2) and phosphor-S6 protein (serines 235/236 and 240/244) antibodies (Cell Signaling). Primary antibodies were used at 1:2000, except for AC40 (1:4000). For mutant huntingtin analysis, dissected brain regions were homogenized in 20 volumes of 20 mM TRIS (pH 7.2), 150 mM sodium chloride, 1 mM EDTA, 1 mM DTT and HALT protease inhibitor cocktail (Pierce) using a Pellet pestle^® ^(Kontes). Samples were cleared at 18000 g for 15 minutes at 4°C. Thirty μg protein was resolved by SDS-PAGE and transferred to PVDF. Membranes were blocked in 5% milk powder in TRIS-buffered saline containing 1% Tween-20 (TBST) and then probed with primary antibody overnight in blocking buffer at 4°C. After washing and incubation in HRP-conjugated secondary antibody membranes were developed using Western Lightening™ chemiluminescent reagent plus (Perkin-Elmer). For analyses of all other proteins, the procedure was identical to that described above except for the homogenization buffer; this comprised 25 mM HEPES (pH 7.4), 75 mM sodium chloride, 12.5 mM β-glycerophosphate, 12.5 mM sodium fluoride, 2.5 mM EGTA, 0.5 mM EDTA, 7.5 mM sodium pyrophosphate, 2 mM sodium vanadate, 0.1% Nonidet-P40 and HALT protease inhibitor. For muscle analysis, the same procedures were used except that tissues were homogenized using a Tissuemiser^® ^(Fisher). For brain, actin was used to normalize Western blots. For muscle, we used parallel run coomassie gels because we do not have a validated housekeeping gene for R6/2 muscle and because actin mRNA is down regulated in this tissue [32].

### Rota-Rod Analysis

Rota-Rod endurance was assessed using an accelerating Rota-rod (Stoelting). The rod speed accelerated from 4.5-45 rpm at a constant rate. Measurements were first taken prior to dosing at 3.5 weeks, then at every 2 weeks of age. For each time point, mice were evaluated on four consecutive days. Day 1 was a training day. On days 2-4 accelerating Rota-rod endurance was evaluated once/day up to a maximum of 15 minutes and endurance times recorded. The average of three trials was used for statistical analysis.

### Histology

At 12 weeks of age, mice were deeply anesthetized with a tribromoethanol-based anesthetic. They were then perfused with freshly prepared room temperature 2% paraformaldehyde in 0.1 M phosphate buffer (pH 7.4) for 15 minutes at a flow rate of 12 mls/minute. Perfused mice were stored at 4°C for 2 hours prior to brain removal. Brains were post-fixed in the same fixative overnight at 4°C prior to cryoprotection for 3 days in 10% glycerol, 2% DMSO and 0.1 M phosphate buffer. The entire striatum was sectioned coronally at 50 μm and every eighth section was mounted and stained for Nissl substance using the thionin method.

### Immunostaining

Fifty-μm sections at the level of the anterior commissure were used for immunohistochemical (IHC) staining for mutant huntingtin aggregates and LAMP1 immunofluorescence (IF). For IHC, we used a 1:2000 dilution of EM48 antibody incubated with floating sections in PBS containing 0.5% Tween-20 for 48 hours to aid penetrance. After washing, sections were incubated in a biotinylated anti-rabbit antibody overnight. Reactivity was developed using the Vectastain ABC kit (Vector Laboratories). Sections were mounted in aqueous medium (Fluoromount G, Southern Biotech) to prevent z-axis shrinkage. For IF, sections were incubated in 1:100 anti-LAMP1 (BioLegend) in PBS containing 0.1% Tween-20 and 10% normal goat serum for 48 hours. Sections were washed three times in PBS, then incubated in AlexaFluor-488 labeled anti-rat antibody (Invitrogen) for 4-hours at 25°C. After washing in PBS, sections were stained with the nucleic acid stain ethidium dimer at 5 μM for 2 hours in the dark. Sections were washed in PBS before mounting. Three-layered z-stack images were collected using a Leica TCS SL confocal microscope. Neurons were identified within the central stack using ethidium dimer signal that delineates nuclei and cytosol. LAMP1 signal was quantified by outlining neuronal cell body outlines and quantifying fluorescence using Metamorph software (Molecular Devices).

### Stereology-

The methodologies used were exactly the same as we have fully described previously [37].

### Biochemical quantification of mutant huntingtin levels

Soluble mutant huntingtin was quantified by time-resolved FRET as described [25]. In brief, muscle samples were homogenized in 20 volumes of PBS containing 0.4% (v/v) Triton-X100 and protease inhibitor using a Precellys^®^24 (Bertin technologies) for 2 × 30 seconds at 5000 rpm. Homogenates were cleared at 3000 rpm for 10 minutes at 4°C. The supernatant was transferred into a fresh tube and total protein was measured using the BCA-Protein detection kit (Perbio, Cramlington, UK). Brain samples were homogenized in 25 mM HEPES (pH 7.4), 75 mM sodium chloride, 12.5 mM beta-glycerophosphate, 12.5 mM sodium fluoride, 2.5 mM EGTA, 0.5 mM EDTA, 7.5 mM sodium pyrophosphate, 2 mM sodium vanadate, 1 mM dithiothreitol, 0.1% nonidet p40, and 0.1% HALT protease inhibitor. The amount of homogenate needed to reach levels in the linear range of time-resolved FRET detection was determined and resulted in 2 μl of homogenate for brain samples (~12 μg protein/well), and 5 μl of homogenate for muscle samples (~17 μg protein/well). Brain or muscle homogenates were mixed with an antibody solution (5 μl) composed of 2B7-Europium-Cryptate (1 ng) and MW1-d2 (10 ng) dissolved in NaH_2_PO_4 _(50 mM, pH 7.4), NaF (400 mM), BSA (0.1% w/v), and Tween 20, (0.05% v/v) in a low-volume 384-well plate and incubated at 4 degrees centigrade overnight. The final volume was 15 μl. Time-resolved FRET with excitation at 320 nm and emission at 620 and 665 nm was measured using an Envision fluorimeter (Perkin-Elmer). Time-resolved FRET signals are given as the 665/620 nm ratio. Background levels (wild-type) were deducted and values normalized for protein concentration. Results are expressed as a percentage of vehicle treated animals. Aggregated huntingtin was quantified using the recently described agarose gel electrophoresis for resolving aggregates (AGERA) method [26]. Briefly, mouse brain samples were homogenized in 10 volumes (w/v) tris-saline (100 mM Tris, pH 7.4, 150 mM NaCl) and Complete Protease Inhibitor (Roche Diagnostics) by 10 ultrasound pulses with a Branson sonifier and stored at −80°C. For 1.7% agarose gels, 1.7 g agarose (Biorad, #161-3101) was dissolved in 100 mL 375 mmol/L Tris-HCl, pH 8.8 and brought to boiling in a microwave oven. After melting, SDS was added to a final concentration of 0.1% (w/v). Gels were poured on short Biorad DNA Sub Cell™ trays. Samples were diluted 1: 1 into non-reducing Laemmli sample buffer (150 mmol/L Tris-HCl pH 6.8, 33% glycerol, 1.2% SDS and bromophenol blue) and incubated for 20 minutes at 95°C. Two-hundred μg of protein was loaded per AGERA lane. After loading, gels were run in Laemmli running buffer (192 mmol/L glycine, 25 mmol/L Tris-base, 0.1% (w/v) SDS) at 100 V, 2 A until the bromophenol blue running front reached the bottom of the gel. Semi-dry electroblotter model B (Ancos, Højby, Denmark) was used to blot the gels on PDVF membranes (Millipore) at 200 mA for 1 hour. Membranes were then developed using MW8 mouse monoclonal antibodies (3 μg/ml), and aggregate quantification performed by densitometry analysis.

### Statistical analysis

All data was analyzed using SAS version 9.1 software (Cary, NC). Rota-rod and body weight data was analyzed using a mixed-model method that included age by treatment interaction effects. Slice functions and t-tests were used to determine significant differences. All other data was analyzed by one-way analysis of variance (ANOVA) using a generalized-linear model procedure followed by pair-wise comparisons. All p-values < 0.05 were considered significant.

## Competing interests

PP, DB, CB, DH and SF are employed by Novartis Institutes for Biomedical Research, Basel, Switzerland.

## Authors' contributions

JF, TC and VC carried out the protein expression and stereology studies. KD and JK carried out the behavioral experiments and maintained the mice. DH and CB carried out the pharmacokinetic analysis. DB and SF completed the quantification of aggregated and soluble mutant huntingtin. JF, PP, MF and SH designed the experiments and wrote the paper with assistance from all other authors. All authors read and approved the final manuscript.

## Supplementary Material

Additional file 1 **Everolimus treatment has no effect on aggregated mutant huntingtin levels in R6/2 brain and muscle**. Mutant huntingtin aggregates were quantified using the AGERA assay in brain and muscle at 12-weeks after 8-weeks of treatment. There is no effect of everolimus on aggregated mutant huntingtin levels in brain (**A-B**) or muscle (**C-D**) of R6/2 HD mice. Representative gels (**A, C**) and quantification (**B, D**). Bars represent means ± SEM. n = 10-12 Click here for file

## References

[B1] The Huntington's Disease Collaborative Research Group A novel gene containing a trinucleotide repeat that is expanded and unstable on Huntington's disease chromosomes Cell 1993 72 971 983 10.1016/0092-8674(93)90585-E 8458085

[B2] Rideout HJ Lang-Rollin I Stefanis L Involvement of macroautophagy in the dissolution of neuronal inclusions Int J Biochem Cell Biol 2004 36 2551 2562 10.1016/j.biocel.2004.05.008 15325592

[B3] Kegel KB Kim M Sapp E McIntyre C Castano JG Aronin N DiFiglia M Huntingtin expression stimulates endosomal-lysosomal activity, endosome tubulation, and autophagy J Neurosci 2000 20 7268 7278 11007884 10.1523/JNEUROSCI.20-19-07268.2000 PMC6772788

[B4] Klionsky DJ Emr SD Autophagy as a regulated pathway of cellular degradation Science 2000 290 1717 1721 10.1126/science.290.5497.1717 11099404 PMC2732363

[B5] Klionsky DJ Cregg JM Dunn WA Jr Emr SD Sakai Y Sandoval IV Sibirny A Subramani S Thumm M Veenhuis M Ohsumi Y A unified nomenclature for yeast autophagy-related genes Dev Cell 2003 5 539 545 10.1016/S1534-5807(03)00296-X 14536056

[B6] Schroder B Wrocklage C Pan C Jager R Kosters B Schafer H Elsasser HP Mann M Hasilik A Integral and associated lysosomal membrane proteins Traffic 2007 8 1676 1686 10.1111/j.1600-0854.2007.00643.x 17897319

[B7] Ravikumar B Vacher C Berger Z Davies JE Luo S Oroz LG Scaravilli F Easton DF Duden R O'Kane CJ Rubinsztein DC Inhibition of mTOR induces autophagy and reduces toxicity of polyglutamine expansions in fly and mouse models of Huntington disease Nat Genet 2004 36 585 595 10.1038/ng1362 15146184

[B8] Aguib Y Heiseke A Gilch S Riemer C Baier M Schatzl HM Ertmer A Autophagy induction by trehalose counteracts cellular prion infection Autophagy 2009 5 361 369 10.4161/auto.5.3.7662 19182537

[B9] Sarkar S Davies JE Huang Z Tunnacliffe A Rubinsztein DC Trehalose, a novel mTOR-independent autophagy enhancer, accelerates the clearance of mutant huntingtin and alpha-synuclein J Biol Chem 2007 282 5641 5652 10.1074/jbc.M609532200 17182613

[B10] Boland B Kumar A Lee S Platt FM Wegiel J Yu WH Nixon RA Autophagy induction and autophagosome clearance in neurons: relationship to autophagic pathology in Alzheimer's disease J Neurosci 2008 28 6926 6937 10.1523/JNEUROSCI.0800-08.2008 18596167 PMC2676733

[B11] Sarkar S Perlstein EO Imarisio S Pineau S Cordenier A Maglathlin RL Webster JA Lewis TA O'Kane CJ Schreiber SL Rubinsztein DC Small molecules enhance autophagy and reduce toxicity in Huntington's disease models Nat Chem Biol 2007 3 331 338 10.1038/nchembio883 17486044 PMC2635561

[B12] Guertin DA Sabatini DM The pharmacology of mTOR inhibition Sci Signal 2009 2 pe24 10.1126/scisignal.267pe24 19383975

[B13] Dowling RJ Topisirovic I Fonseca BD Sonenberg N Dissecting the role of mTOR: lessons from mTOR inhibitors Biochim Biophys Acta 2010 1804 433 439 20005306 10.1016/j.bbapap.2009.12.001

[B14] Inoki K Corradetti MN Guan KL Dysregulation of the TSC-mTOR pathway in human disease Nat Genet 2005 37 19 24 10.1038/ng1494 15624019

[B15] Sarkar S Ravikumar B Floto RA Rubinsztein DC Rapamycin and mTOR-independent autophagy inducers ameliorate toxicity of polyglutamine-expanded huntingtin and related proteinopathies Cell Death Differ 2009 16 46 56 10.1038/cdd.2008.110 18636076

[B16] Alonso MM Jiang H Yokoyama T Xu J Bekele NB Lang FF Kondo S Gomez-Manzano C Fueyo J Delta-24-RGD in combination with RAD001 induces enhanced anti-glioma effect via autophagic cell death Mol Ther 2008 16 487 493 10.1038/sj.mt.6300400 18253154

[B17] Crazzolara R Bradstock KF Bendall LJ RAD001 (everolimus) induces autophagy in acute lymphoblastic leukemia Autophagy 2009 5 10.4161/auto.5.5.8507 19363300

[B18] Eisen HJ Tuzcu EM Dorent R Kobashigawa J Mancini D Valantine-von Kaeppler HA Starling RC Sorensen K Hummel M Lind JM Everolimus for the prevention of allograft rejection and vasculopathy in cardiac-transplant recipients N Engl J Med 2003 349 847 858 10.1056/NEJMoa022171 12944570

[B19] Motzer RJ Escudier B Oudard S Hutson TE Porta C Bracarda S Grunwald V Thompson JA Figlin RA Hollaender N Efficacy of everolimus in advanced renal cell carcinoma: a double-blind, randomised, placebo-controlled phase III trial Lancet 2008 372 449 456 10.1016/S0140-6736(08)61039-9 18653228

[B20] Mangiarini L Sathasivam K Seller M Cozens B Harper A Hetherington C Lawton M Trottier Y Lehrach H Davies SW Bates GP Exon 1 of the HD gene with an expanded CAG repeat is sufficient to cause a progressive neurological phenotype in transgenic mice Cell 1996 87 493 506 10.1016/S0092-8674(00)81369-0 8898202

[B21] Stack EC Kubilus JK Smith K Cormier K Del Signore SJ Guelin E Ryu H Hersch SM Ferrante RJ Chronology of behavioral symptoms and neuropathological sequela in R6/2 Huntington's disease transgenic mice J Comp Neurol 2005 490 354 370 10.1002/cne.20680 16127709

[B22] Kabeya Y Mizushima N Ueno T Yamamoto A Kirisako T Noda T Kominami E Ohsumi Y Yoshimori T LC3, a mammalian homologue of yeast Apg8p, is localized in autophagosome membranes after processing Embo J 2000 19 5720 5728 10.1093/emboj/19.21.5720 11060023 PMC305793

[B23] Devon RS Orban PC Gerrow K Barbieri MA Schwab C Cao LP Helm JR Bissada N Cruz-Aguado R Davidson TL Als2-deficient mice exhibit disturbances in endosome trafficking associated with motor behavioral abnormalities Proc Natl Acad Sci USA 2006 103 9595 9600 10.1073/pnas.0510197103 16769894 PMC1480452

[B24] Andrejewski N Punnonen EL Guhde G Tanaka Y Lullmann-Rauch R Hartmann D von Figura K Saftig P Normal lysosomal morphology and function in LAMP-1-deficient mice J Biol Chem 1999 274 12692 12701 10.1074/jbc.274.18.12692 10212251

[B25] Weiss A Abramowski D Bibel M Bodner R Chopra V DiFiglia M Fox J Kegel K Klein C Grueninger S Single-Step Detection of Mutant Huntingtin in Animal and Human Tissues: a BioAssay for Huntington's Disease Anal Biochem 2009 395 8 15 10.1016/j.ab.2009.08.001 19664996

[B26] Weiss A Klein C Woodman B Sathasivam K Bibel M Regulier E Bates GP Paganetti P Sensitive biochemical aggregate detection reveals aggregation onset before symptom development in cellular and murine models of Huntington's disease J Neurochem 2008 104 846 858 17986219 10.1111/j.1471-4159.2007.05032.x

[B27] Tanaka M Machida Y Niu S Ikeda T Jana NR Doi H Kurosawa M Nekooki M Nukina N Trehalose alleviates polyglutamine-mediated pathology in a mouse model of Huntington disease Nat Med 2004 10 148 154 10.1038/nm985 14730359

[B28] Ballou LM Lin RZ Rapamycin and mTOR kinase inhibitors J Chem Biol 2008 1 27 36 10.1007/s12154-008-0003-5 19568796 PMC2698317

[B29] Chugh BP Lerch JP Yu LX Pienkowski M Harrison RV Henkelman RM Sled JG Measurement of cerebral blood volume in mouse brain regions using micro-computed tomography Neuroimage 2009 47 1312 1318 10.1016/j.neuroimage.2009.03.083 19362597

[B30] Schuler W Sedrani R Cottens S Haberlin B Schulz M Schuurman HJ Zenke G Zerwes HG Schreier MH SDZ RAD, a new rapamycin derivative: pharmacological properties in vitro and in vivo Transplantation 1997 64 36 42 10.1097/00007890-199707150-00008 9233698

[B31] Kosinski CM Schlangen C Gellerich FN Gizatullina Z Deschauer M Schiefer J Young AB Landwehrmeyer GB Toyka KV Sellhaus B Lindenberg KS Myopathy as a first symptom of Huntington's disease in a Marathon runner Mov Disord 2007 22 1637 1640 10.1002/mds.21550 17534945

[B32] Luthi-Carter R Hanson SA Strand AD Bergstrom DA Chun W Peters NL Woods AM Chan EY Kooperberg C Krainc D Dysregulation of gene expression in the R6/2 model of polyglutamine disease: parallel changes in muscle and brain Hum Mol Genet 2002 11 1911 1926 10.1093/hmg/11.17.1911 12165554

[B33] Bodine SC Stitt TN Gonzalez M Kline WO Stover GL Bauerlein R Zlotchenko E Scrimgeour A Lawrence JC Glass DJ Yancopoulos GD Akt/mTOR pathway is a crucial regulator of skeletal muscle hypertrophy and can prevent muscle atrophy in vivo Nat Cell Biol 2001 3 1014 1019 10.1038/ncb1101-1014 11715023

[B34] Ferrante RJ Kubilus JK Lee J Ryu H Beesen A Zucker B Smith K Kowall NW Ratan RR Luthi-Carter R Hersch SM Histone deacetylase inhibition by sodium butyrate chemotherapy ameliorates the neurodegenerative phenotype in Huntington's disease mice J Neurosci 2003 23 9418 9427 14561870 10.1523/JNEUROSCI.23-28-09418.2003 PMC6740577

[B35] Schilling G Becher MW Sharp AH Jinnah HA Duan K Kotzuk JA Slunt HH Ratovitski T Cooper JK Jenkins NA Intranuclear inclusions and neuritic aggregates in transgenic mice expressing a mutant N-terminal fragment of huntingtin Hum Mol Genet 1999 8 397 407 10.1093/hmg/8.3.397 9949199

[B36] Crazzolara R Cisterne A Thien M Hewson J Baraz R Bradstock KF Bendall LJ Potentiating effects of RAD001 (Everolimus) on vincristine therapy in childhood acute lymphoblastic leukemia Blood 2009 113 3297 3306 10.1182/blood-2008-02-137752 19196656

[B37] Chopra V Fox JH Lieberman G Dorsey K Matson W Waldmeier P Housman DE Kazantsev A Young AB Hersch S A small-molecule therapeutic lead for Huntington's disease: preclinical pharmacology and efficacy of C2-8 in the R6/2 transgenic mouse Proc Natl Acad Sci USA 2007 104 16685 16689 10.1073/pnas.0707842104 17925440 PMC2034257

