# Supplementary figures and images for: The mTOR kinase inhibitor Everolimus decreases S6 kinase phosphorylation but fails to reduce mutant huntingtin levels in brain and is not neuroprotective in the R6/2 mouse model of Huntington's disease
Source: Mol Neurodegener. 2010 Jun 22;5:26. doi: 10.1186/1750-1326-5-26 (PMC2908080; doi:10.1186/1750-1326-5-26)

Figure S1

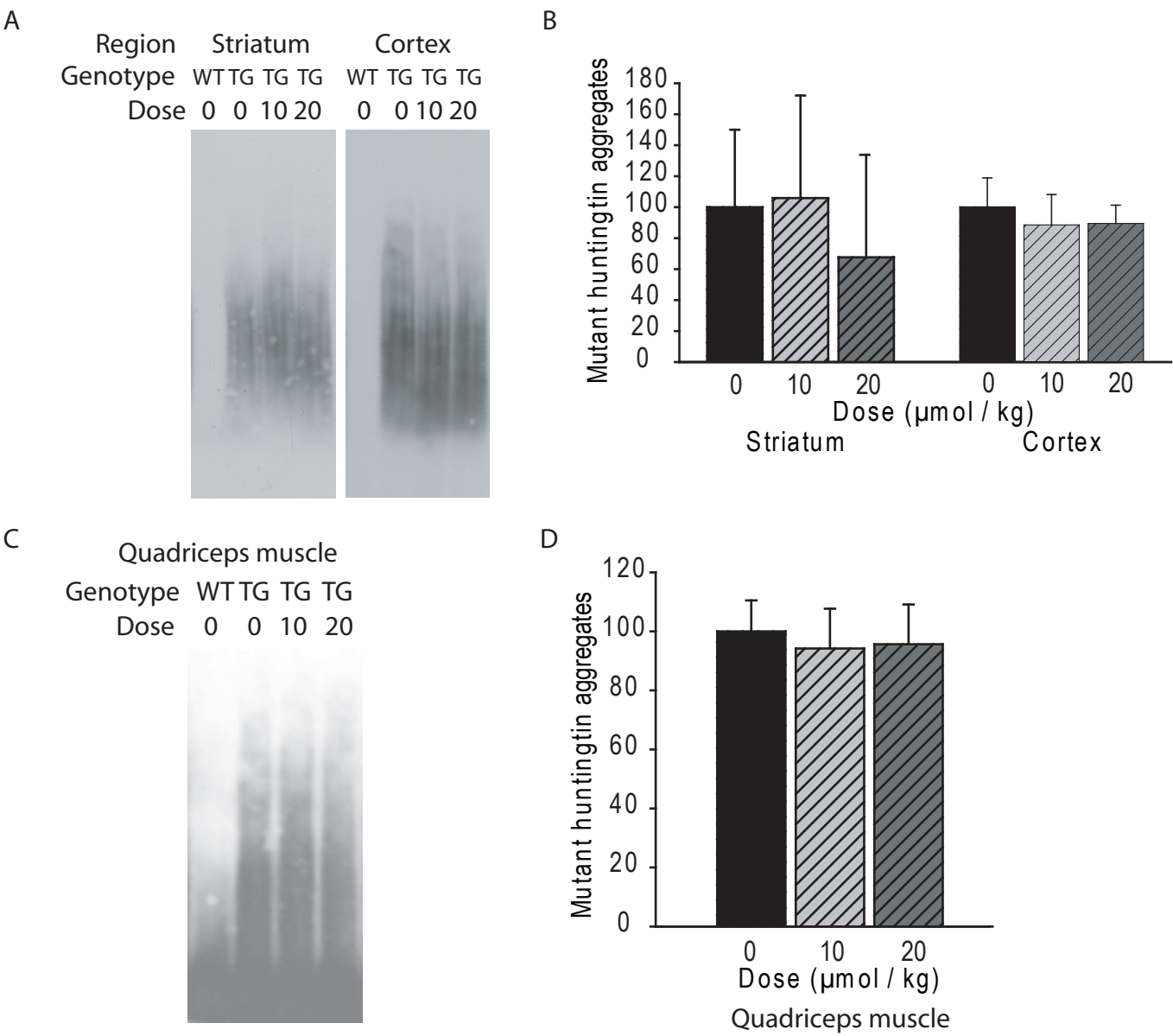

Supplement: Additional file 1 — Everolimus treatment has no effect on aggregated mutant huntingtin levels in R6/2 brain and muscle. Mutant huntingtin aggregates were quantified using the AGERA assay in brain and muscle at 12-weeks after 8-weeks of treatment. There is no effect of everolimus on aggregated mutant huntingtin levels in brain (A-B) or muscle (C-D) of R6/2 HD mice. Representative gels (A, C) and quantification (B, D). Bars represent means ± SEM. n = 10-12 [file 1750-1326-5-26-S1.PDF]
